# Polygalae Radix Extract Prevents Axonal Degeneration and Memory Deficits in a Transgenic Mouse Model of Alzheimer’s Disease

**DOI:** 10.3389/fphar.2017.00805

**Published:** 2017-11-14

**Authors:** Tomoharu Kuboyama, Keisuke Hirotsu, Tetsuya Arai, Hiroo Yamasaki, Chihiro Tohda

**Affiliations:** ^1^Division of Neuromedical Science, Institute of Natural Medicine, University of Toyama, Toyama, Japan; ^2^R&D Center, Kobayashi Pharmaceutical Co., Ltd., Ibaraki, Japan

**Keywords:** Alzheimer’s disease, Polygalae Radix, axon degeneration, amyloid β, endocytosis, growth cone collapse, 5XFAD mice

## Abstract

Memory impairments in Alzheimer’s disease (AD) occur due to degenerated axons and disrupted neural networks. Since only limited recovery is possible after the destruction of neural networks, preventing axonal degeneration during the early stages of disease progression is necessary to prevent AD. Polygalae Radix (roots of *Polygala tenuifolia*; PR) is a traditional herbal medicine used for sedation and amnesia. In this study, we aimed to clarify and analyze the preventive effects of PR against memory deficits in a transgenic AD mouse model, 5XFAD. 5XFAD mice demonstrated memory deficits at the age of 5 months. Thus, the water extract of Polygalae Radix (PR extract) was orally administered to 4-month-old 5XFAD mice that did not show signs of memory impairment. After consecutive administrations for 56 days, the PR extract prevented cognitive deficit and axon degeneration associated with the accumulation of amyloid β (Aβ) plaques in the perirhinal cortex of the 5XFAD mice. PR extract did not influence the formation of Aβ plaques in the brain of the 5XFAD mice. In cultured neurons, the PR extract prevented axonal growth cone collapse and axonal atrophy induced by Aβ. Additionally, it prevented Aβ-induced endocytosis at the growth cone of cultured neurons. Our previous study reported that endocytosis inhibition was enough to prevent Aβ-induced growth cone collapse, axonal degeneration, and memory impairments. Therefore, the PR extract possibly prevented axonal degeneration and memory impairment by inhibiting endocytosis. PR is the first preventive drug candidate for AD that inhibits endocytosis in neurons.

## Introduction

Alzheimer’s disease is a progressive, degenerative, and irreversible neurological disorder. Although several clinical drugs are available for AD patients, these drugs only moderate the progression of AD. None of the AD treatments have succeeded in recovering cognitive function once the disease has progressed. Thus, since it is too late to treat AD with severe symptoms, a therapy or the prevention of AD at an early stage or before the onset of symptoms is required. Memory deficits are one of the most important core features of AD. Although AD can develop due to heterogeneous risk factors, such as genome, epigenome, and environments (Killin et al., 2016; Maloney and Lahiri, 2016; Pimenova et al., 2017), the common features of AD include amyloid β (Aβ)-induced degeneration of neurites, disruption of neural networks, and memory deficits (Dickson and Vickers, 2001; Hardy and Selkoe, 2002; Perl, 2010; Selkoe and Hardy, 2016). Preventing axonal degeneration could inhibit the progression of memory deficits in AD (Dickson and Vickers, 2001; Hardy and Selkoe, 2002; Simmons et al., 2014). Additionally, axonal regeneration is related to recovery from cognitive dysfunction in murine models of AD (Tohda, 2016).

Polygalae Radix (roots of *Polygala tenuifolia*; PR) is a traditional herbal medicine, which has been clinically used for memory loss in East Asia (Wu et al., 2011; May et al., 2013). There are many reports about the anti-AD properties of PR. For example, PR extracts and their constituents were reported to have a protective effect in cultured neurons from Aβ-induced neurotoxicity (Xu et al., 2011); they enhanced axon elongation when cultured neurons were already undergoing Aβ-induced axon atrophy (Naito and Tohda, 2006), inhibited Aβ secretion in cultured cells (Jia et al., 2004; Lv et al., 2009), and recovered memory in intrahippocampally Aβ-injected mice (Xu et al., 2011; Liu et al., 2015). Additionally, BT-11, an extract of PR, was reported to recover memory in stress- or scopolamine-induced amnestic rats (Park et al., 2002; Shin et al., 2009), and enhance memory in healthy humans (Lee et al., 2009). Taking these reports into consideration, PR may also have preventive effects against AD. At present, however, there are no studies that have demonstrated that PR prevents the development of AD in humans or animal models.

In AD, the increases in the level of Aβ in the brain precede memory deficits (Jack et al., 2010). A similar phenomenon is also observed in a transgenic mouse model of AD, 5XFAD mice, which expresses mutant human amyloid precursor protein (the Swedish mutations: K670N and M671L; the Florida mutation: I716V; and the London mutation: V717I) and PS1 (M146L; L286V) transgenes, under the neuron-specific mouse Thy-1 promoter (Oakley et al., 2006). Aβ plaques accumulate in the brains of 5XFAD transgenic mice by the age of 2 months, and memory impairments by 4–5 months (Oakley et al., 2006) and axon degeneration by 4–7 months (Tohda et al., 2011, 2012; Yang et al., 2017). This corroborated with the trend seen in patients with AD (Benes et al., 1991; Masliah et al., 1993). In the current study, PR water extract was consecutively administered before the onset of memory impairments in the 5XFAD mice, and its preventive effects on axonal degeneration and memory impairment were investigated. We focused on endocytosis as its inhibition is sufficient to prevent Aβ-induced axonal degeneration and memory deficits, and cause these preventive effects (Kuboyama et al., 2015).

## Materials and Methods

### Polygalae Radix Extract

The dried powder of the water extract of Polygalae Radix (PR extract; Management No. Q14504) was obtained from Kobayashi Pharmaceutical Co., Ltd. (Ibaraki, Japan). To check the quality of the PR extract, the concentrations of two compounds, i.e., tenuifolin and 3′,6-di-*O*-sinapoyl sucrose ester, were measured using high performance liquid chromatography (HPLC) analysis according to Hong Kong Chinese Materia Medica Standards^1^. These two compounds are index components, which are used to check the quality of PR, according to the Hong Kong Chinese Materia Medica Standards and China Pharmacopoeia (Chinese Pharmacopoeia Commission, 2015). For the quantification of tenuifolin, the PR extract (0.15 g) was dissolved in 10% sodium hydroxide (30 mL; Cat. No. 198-13765; Wako, Osaka, Japan), and the solution was heated and refluxed at 110°C for 90 min. The solution was adjusted to pH 4–5 by adding hydrochloric acid (Cat. No. 080-01066; Wako). Water was added to bring the solution up to 100 mL, and 30 mL of the solution was extracted with 1-butanol (50 mL; Cat. No. 026-03326; Wako) twice. The 1-butanol solution was dried, and then resolubilized with methanol (10 mL; Cat. No. 132-06471; Wako). A total of 10 μL of the methanol solution was applied (Shimadzu, Kyoto, Japan) to an HPLC system, on a YMC-Pack ODS-AM AM-312 (6.0 mm i.d. × 150 mm, YMC, Kyoto, Japan) column held at 30°C with a flow rate of 1 mL min^-1^, and was detected using a photodiode array detector (SPD-M20A, Shimadzu). In the mobile phase, 0.05% phosphoric acid (v/v) (Cat. No. 167-02166; Wako) and acetonitrile (Cat. No. 015-08633; Wako) (33:17) were used. To quantify 3′,6-di-*O*-sinapoyl sucrose ester, the PR extract (0.3 g) was dissolved in 50% methanol (50 mL). A total of 10 μL of the 50% methanol solution was applied to the HPLC system on a Cosmosil 5C18-AR-II (4.6 mm i.d. × 150 mm, nacalai tesque, Kyoto, Japan) column held at 30°C with a flow rate of 1 mL min^-1^. Water, acetonitrile, and formic acid (Cat. No. 063-5895; Wako) (860:160:1) were used in the mobile phase. Standard curves were produced to measure concentrations of tenuifolin and 3′,6-di-*O*-sinapoyl sucrose ester in the PR extract. These two compounds were purchased from TOKIWA phytochemical Co., Ltd. (Sakura, Japan) (tenuifolin, Cat. No. P2515; 3′,6-di-*O*-sinapoyl sucrose ester, Cat. No. P2939). HPLC profiles of the PR extract are shown in **Supplementary Figure S1**. Concentrations of tenuifolin and 3′,6-di-*O*-sinapoyl sucrose ester in the PR extract were 2.89 and 0.86%, respectively.

### Animal Experiments

All animal experiments were conducted in accordance with the Guidelines for the Care and Use of Laboratory Animals at the Sugitani Campus of the University of Toyama. The Committee for Animal Care and Use at the Sugitani Campus of the University of Toyama approved all protocols. The approval number for the animal experiments is A2014INM-1 and A2017INM-1, and the confirmation number for the recombinant gene experiments is G2013INM-1.

A novel object recognition test was performed using AD model 5XFAD mice or wild-type (WT) mice (4-month-old, male and female mice; Jackson laboratory, Bar Harbor, ME, United States) as described previously (Kuboyama et al., 2015). Briefly, each mouse was trained to habituate to two identical objects in an open box for 10 min, and were then returned to their home cage. The test session was the performed for 10 min, 22 h later. In the test session, one of the objects was replaced with a novel object in the same place. The number of times the mouse encountered each object was counted. Preference indices were calculated.

After the test session finished, the PR extract (at a dose of 12 or 60 mg kg^-1^ day^-1^) or a vehicle solution (sterilized saline) was orally administered, daily, to the mice for 56 days. Saline is an innocuous medium, and treatment with saline is akin to an invasive-free treatment. The day after the last administration, a second trial of the novel object recognition test was performed using different new objects.

### Immunohistochemistry

After the second trial of the novel object recognition test finished, the mice were euthanized and perfusion-fixed with 4% paraformaldehyde (PFA; Cat. No. 162-16065; Wako) dissolved in phosphate buffered saline (PBS). Brains were cryoprotected in 30% sucrose and frozen on dry ice. Frozen brains were coronally sectioned at 14 μm and further fixed with 4% PFA at 4°C overnight. The sections were then washed with PBS and incubated with blocking solution, i.e., 5% normal goat serum (NGS; Cat. No. 143-06561; Wako) in a solution of 0.3% Triton X-100 (Cat. No. 168-11805; Wako) and PBS for 1 h at room temperature. Then, sections were incubated in a solution containing anti-phosphorylated neurofilament-H (pNF-H) monoclonal antibody (an axonal marker; dilution 1:500; clone SMI-35; Covance, Dedham, MA, United States), anti-Aβ polyclonal antibody (dilution 1:1000; Cat. No. AB5076; Millipore, Billerica, MA, United States), and 1% BSA (Cat. No. 010-25783; Wako) in a solution of 0.3% Triton X-100 and PBS at 4°C overnight. Next, the sections were incubated in a solution containing Alexa Fluor 594-conjugated goat anti-mouse IgG (dilution 1:400; Cat. No. A-11005; Thermo Fisher Scientific, Waltham, MA, United States), Alexa Fluor 488-conjugated goat anti-rabbit IgG (dilution 1:400; Cat. No. A-11008; Thermo Fisher Scientific), and 1% bovine serum albumin (BSA) in a solution of 0.3% Triton X-100 and PBS at room temperature for 2 h. A series of staining procedures was performed using the Shandon Sequenza slide rack (Thermo Fisher Scientific). Fluorescence images (325 μm × 426 μm) were captured using a 20× NA 0.8 dry objective lens (Plan-Apochromat, Carl Zeiss, Oberkochen, Germany) and a charge-coupled device camera (AxioCam MRm, binning set at 1 × 1, Carl Zeiss) on an inverted microscope (AxioObserver Z1, Carl Zeiss). A total of six images were captured for each brain region in each mouse. The density of Aβ plaques in each brain region and densities of degenerated axons merged on the Aβ plaque were quantified using ImageJ software (National Institutes of Health, Bethesda, MD, United States) as described previously (Tohda et al., 2012).

### Collapse Assay

Cerebral cortical neurons (embryonic day 14; ddY mice; SLC, Hamamatsu, Japan) were cultured in 8-well chamber slides (Falcon, Franklin Lakes, NJ, United States) as described previously (Kuboyama et al., 2015). Three days after the culture started, the neurons were treated with either the PR extract (10 and 100 μg mL^-1^) or a vehicle solution (distilled water) for 30 min. Then, they were treated with either full length Aβ1–42 (1 μM; Sigma, St. Louis, MO, United States) or active partial fragment Aβ25—35 (10 μM; Sigma) for 1 h, and fixed with 4% PFA and 4% sucrose in PBS at 37°C for 1 h. Aβ1–42 and Aβ25–35 were aggregated before the treatment as described previously (Kuboyama et al., 2015). The most effective doses of Aβ1–42 and Aβ25–35 were selected based on our previous study (Kuboyama et al., 2015). The entire area in each chamber (7.8 mm × 9 mm) was automatically captured with a 20× dry objective lens (PlanApo λ, Keyence, Osaka, Japan) on an inverted microscope (BZ-710, Keyence). Collapse scores were quantified as described previously (Kuboyama et al., 2015). Briefly, the longest neurite in each neuron was judged as an axon, and growth cones (axonal endings) lacking lamellipodia or possessing fewer than three filopodia were judged as collapsed growth cones (Dotti et al., 1988; Jurney et al., 2002). The collapsed growth cones were scored 1 point, while healthy growth cones were scored 0 point. An average of the scores in each treatment group was shown as a collapse score.

### Axonal Growth Assay

After cortical neurons were cultured for 3 days, Aβ25–35 (10 μM) was simultaneously treated with PR extract (at doses of 0.1, 1, 10, and 100 μg mL^-1^) for 4 days. After that, neurons were fixed with 4% PFA in PBS and blocked with 5% BSA in a solution of 0.3% Triton X-100 and PBS for 1 h at room temperature. The neurons were then incubated in a solution containing anti-pNF-H monoclonal antibody (dilution 1:500), anti-microtubule associated protein 2 polyclonal antibody (a neuronal marker; dilution 1:1000; Cat. No. ab32454; Abcam, Cambridge, United Kingdom), and 5% BSA in a solution of 0.3% Triton X-100 and PBS at 4°C overnight. Next, the neurons were incubated in a solution containing Alexa Fluor 594-conjugated goat anti-mouse IgG (dilution 1:400), Alexa Fluor 488-conjugated goat anti-rabbit IgG (dilution 1:400), DAPI (1 μg mL^-1^, Sigma), and 3% BSA and 1% NGS in a solution of 0.3% Triton X-100 and PBS for 2 h at room temperature. Fluorescence images (650 μm × 852 μm) were captured using a 10× NA 0.4 dry objective lens on an inverted microscope (AxioObserver Z1). The lengths of axons per neuron were measured using MetaMorph 7.8 software (Molecular Devices, Sunnyvale, CA, United States).

### Endocytosis Experiments

Three days after the cortical neurons were cultured, they were treated with either the PR extract (100 μg ml^-1^) or a vehicle solution (distilled water) for 30 min. After that, FM1-43FX (20 μM; Thermo Fisher Scientific) was added to the culture medium, and Aβ1–42 (1 μM) or vehicle solution (distilled water) was added 1 min later. The neurons were then washed twice with warmed culture medium 20 min later and fixed with 4% PFA and 4% sucrose in PBS for 1 h at 37°C. Images of the growth cones were captured using a 63× NA 1.40 oil imersion objective lens on an inverted microscope (AxioObserver Z1). A total of 50 images were captured for each treatment group. The density of the FM1-43FX-positive area in the normal growth cones were quantified using ImageJ software as described previously (Kuboyama et al., 2015).

### Statistics

Statistical comparisons were performed using a paired (two-tailed) *t*-test, one-way ANOVA followed by a *post hoc* Bonferroni’s multiple comparison test, Kruskal–Wallis test followed by a *post hoc* Dunn’s multiple comparison test, or two-way repeated measures ANOVA. Statistical analyses were performed using Prism 5.04 software (GraphPad software, San Diego, CA, United States). Statistical significance was set at *P* < 0.05. The effect sizes (*d*) were calculated using the G^∗^power 3 statistical software (Faul et al., 2007).

## Results

### PR Extract Prevents Memory Impairment in 5XFAD Mice

**FIGURE 1:**
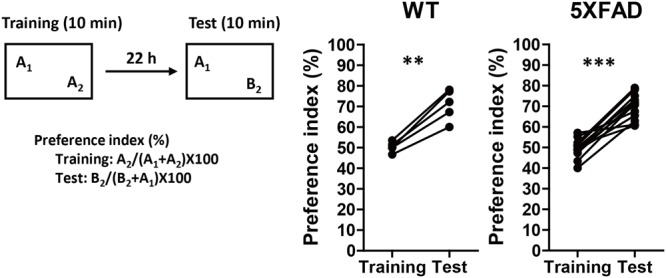
Memory function in 5XFAD mice before the administration of Polygalae Radix (PR) extract. Novel object recognition tests were performed using 4-month-old wild-type (WT) and 5XFAD mice. Preference indices of objects A_2_ (training session) and B_2_ (test session) were quantified. The values of preference indices of each mouse are shown. ^∗∗^*P* < 0.01, ^∗∗∗^*P* < 0.001, paired *t*-test, *n* = 5–14 mice.

5XFAD mice, which have previously shown various AD-like phenotypes, such as Aβ deposition, phosphorylated tau deposition, neuronal loss, axonal degeneration, and memory deficits (Oakley et al., 2006; Urano and Tohda, 2010; Joyashiki et al., 2011; Tohda et al., 2011, 2012) were used to model AD in the current study. PR was administered before the onset of memory deficits to investigate its preventive effects against AD. The age at which 5XFAD mice showed signs of memory impairments was determined using 4-month-old 5XFAD or WT mice that underwent a novel object recognition test (**Figure 1**). In the training session, both groups equally explored each object; the preference indices were approximately 50%. In the test session, WT and 5XFAD mice significantly increased the time spent exploring the replaced novel object (*P* = 0.0012, *d* = 3.71, and *P* < 0.0001, *d* = 2.80, respectively). Mice normally prefer a novel object. Therefore, **Figure 1** demonstrates that 5XFAD mice and WT mice remembered the familiar object and preferred the novel object. Since cognitive function in the 5XFAD and WT mice seemed to be normal at this age, it was deemed that the cognitive function in the 5XFAD mice was likely to be normal until the age of 4 months. The onset of memory deficits occurred at 5 months of age (**Supplementary Figure S2A**).

**FIGURE 2:**
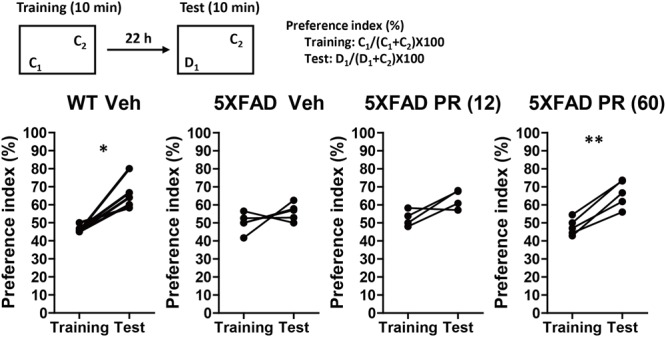
Effects of Polygalae Radix (PR) extract on memory deficits in 5XFAD mice. Vehicle solution (Veh) or PR extract [at doses of 12 or 60 mg kg^-1^ day^-1^; PR (12), PR (60), respectively] were orally administered daily for 56 days to 4-month-old WT or 5XFAD mice. A novel object recognition test was performed the day after the last administration. Preference indices of objects C_1_ (training session) and D_1_ (test session) were quantified. The values of preference indices of each mouse are shown. ^∗^*P* < 0.05, ^∗∗^*P* < 0.01; paired *t*-test, *n* = 4–5 mice.

The PR extract or vehicle solution was administered daily to the 4-month-old WT or 5XFAD mice for 56 days. At the age of 6 months, a second trial of the novel object recognition test was performed (**Figure 2**). There was a significant increase in the preference index in the vehicle-administered WT mice during the test session (*P* = 0.011, *d* = 2.00), whereas there was no increase in the vehicle-administered 5XFAD mice (*P* = 0.26, *d* = 0.58). This finding suggested that memory deficits occurred in 6-month-old 5XFAD mice. In contrast, the administration of a high dose of the PR extract (60 mg kg^-1^ day^-1^) significantly increased the preference index in the test session for the 5XFAD mice (*P* = 0.0016, *d* = 3.43). The administration of a low dose (12 mg kg^-1^ day^-1^) of the PR extract showed a tendency to increase the preference index in the test session (*P* = 0.080, *d* = 1.31). These results demonstrated that the PR extract prevented impairment of object recognition memory in the 5XFAD mice. The administration of PR extract did not influence body weight or exploratory behaviors of the mice (**Supplementary Figure S3**).

### PR Prevents Axonal Degeneration in the Brain of 5XFAD Mice

**FIGURE 3:**
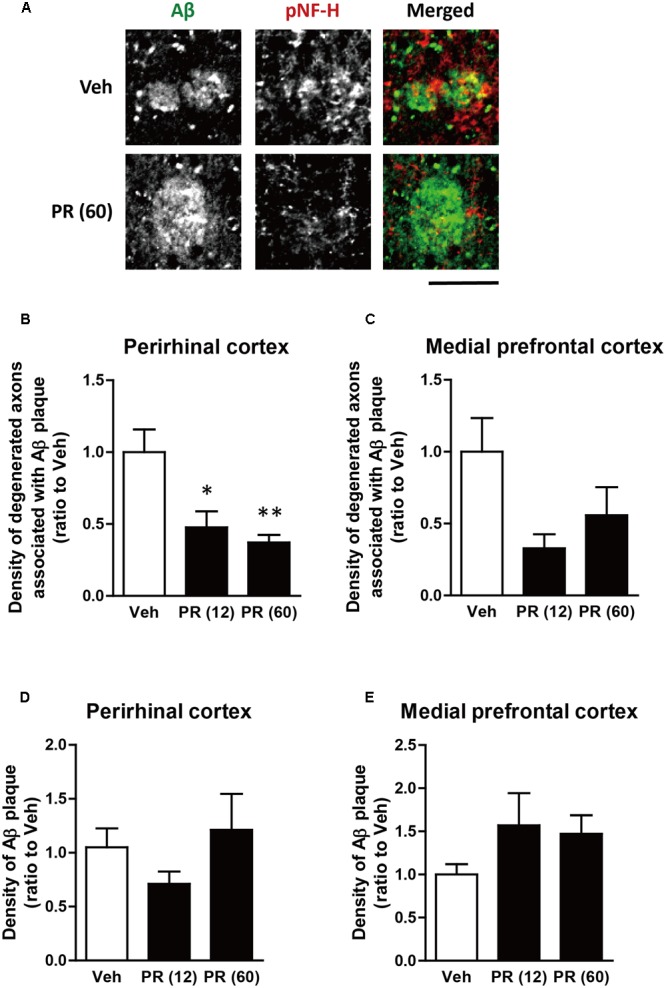
Effects of Polygalae Radix (PR) extract on amyloid β (Aβ) plaques and degenerated axons in 5XFAD mice. Vehicle solution (Veh) or PR extract [at doses of 12 or 60 mg kg^-1^ day^-1^, PR (12), PR (60), respectively] were orally administered daily for 56 days to 4-month-old wild-type (WT) or 5XFAD mice. The mice were euthanized 2 days after the last administration, and the brains were sectioned and immunostained for phosphorylated neurofilament-H (pNF-H, an axonal marker, red) and Aβ (green). **(A)** Representative fluorescence images of the perirhinal cortex are shown. Scale bar, 10 μm. **(B,C)** The density of degenerated axons associated with the Aβ plaques was quantified in the perirhinal cortex **(B)** and medial prefrontal cortex **(C)**. **(D,E)** The density of Aβ plaques was quantified in the perirhinal cortex **(D)** and medial prefrontal cortex **(E)**. The mean values of the data are presented together with the standard error. ^∗^*P* < 0.05, ^∗∗^*P* < 0.01 vs. Veh; Bonferroni’s multiple comparison test, *n* = 4–5 mice.

After behavioral experiments, brain tissue was extracted from mice and the expression of pNF-H (an axonal marker) and Aβ in the perirhinal and medial prefrontal cortices of 5XFAD mice were evaluated using immunohistochemistry. Aβ plaques were observed in both regions and bulb-like degenerated axons overlapped the plaques (**Figure 3**). This was observed in regions related to memory functions (Barker and Warburton, 2011; Vilberg and Davachi, 2013; Tanimizu et al., 2017). We previously confirmed that Aβ plaques and degenerated axons were observed in these regions of 5XFAD mice (Tohda et al., 2011, 2012; Yang et al., 2017), which corroborated with findings from patients with AD (Benes et al., 1991; Masliah et al., 1993). The administration of PR extract (at doses of 12 or 60 mg kg^-1^ day^-1^) significantly decreased the density of degenerated axons associated with the plaques in the perirhinal cortex (**Figures 3A,B**). Although PR extract administration (at doses of 12 and 60 mg kg^-1^ day^-1^) also decreased the density of degenerated axons in the medial prefrontal cortex, this change was not significant (**Figure 3C**). The density of Aβ deposits in the perirhinal and prefrontal cortices remained unchanged following the administration of PR extract (at doses of 12 and 60 mg kg^-1^ day^-1^) (**Figures 3D,E**). Therefore, in 5XFAD mice, the PR extract partially prevented axonal degeneration and cognitive impairment, without affecting Aβ deposition in the brain.

### PR Inhibits Aβ-Induced Axonal Collapse and Endocytosis

**FIGURE 4:**
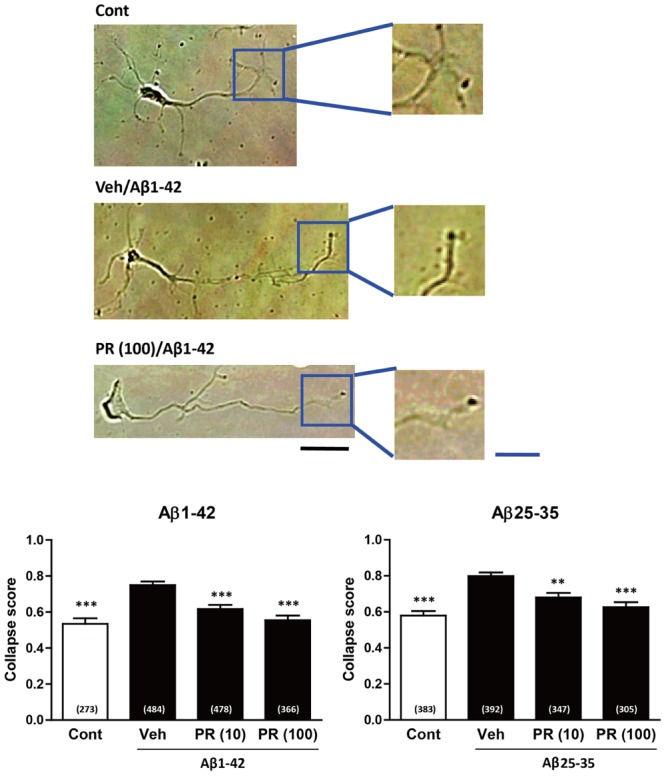
Effects of the Polygalae Radix (PR) extract on amyloid β (Aβ)-induced growth cone collapse. Cortical neurons were cultured for 3 days, and treated for 30 min with vehicle solution (Cont, Veh) or PR extract [at doses of 10 or 100 μg ml^-1^; PR (10), PR (100), respectively]. Next, they were treated with vehicle solution (Cont), Aβ1–42 (1 μM), or Aβ25–35 (10 μM) for 1 h. The neurons were then fixed. Representative bright field images with oblique illumination are shown **(upper)**. Black scale bar, 20 μm. Blue scale bar, 10 μm. The growth cone shapes were quantified as collapse scores. The mean values of the data are presented together with the standard error **(lower)**. The numbers in parentheses indicate the measured numbers of growth cones. ^∗∗^*P* < 0.01, ^∗∗∗^*P* < 0.001 vs. Veh/Aβ; Dunn’s multiple comparison test.

**FIGURE 5:**
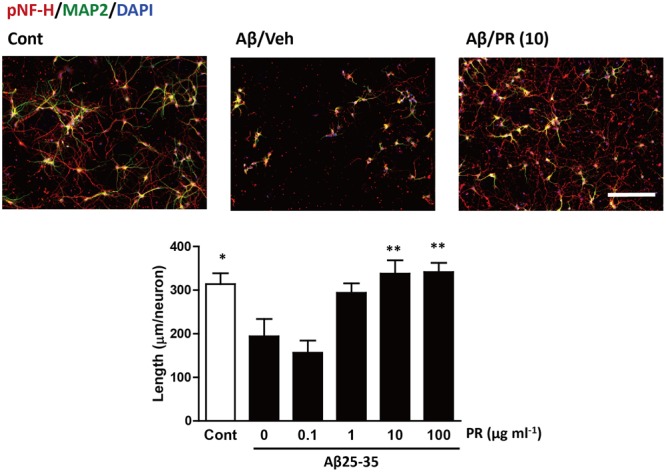
Effects of Polygalae Radix (PR) extract on amyloid β (Aβ)-induced axonal atrophy. Cortical neurons were cultured for 3 days and treated simultaneously with vehicle solution (Cont) or Aβ25–35 (10 μM), and vehicle solution (Cont, 0 μg ml^-1^) or PR extract (at doses of 0.1, 1, 10, or 100 μg ml^-1^) for 4 days. Thereafter, the neurons were fixed and immunostained for phosphorylated neurofilament-H (pNF-H, an axonal marker, red) and microtubule associated protein 2 (MAP2, a neuronal marker, green). DAPI (blue) was used for counterstaining. Representative fluorescence images are shown **(upper)**. Scale bar, 200 μm. The total axonal length per neuron were quantified. The mean values of the data are presented together with the standard error **(lower)**. ^∗∗^*P* < 0.01 vs. 0 μg/mL PR + Aβ25–35; Bonferroni’s multiple comparison test, *n* = 10 images.

Degeneration of axonal endings (axonal collapse) is an early event induced by Aβ in cultured neurons (Kuboyama et al., 2015). Both Aβ1–42 and Aβ25–35 induced axonal collapse in cultured neurons (**Figure 4**) as previously demonstrated (Kuboyama et al., 2015). Aβ25–35 is an active partial fragment of Aβ, which is commonly used as an alternative to full length Aβ1–42 and shows similar pharmacological effects to Aβ1–42, such as neurotoxicity, axonal atrophy, and axonal collapse (Yankner et al., 1990; Kuboyama et al., 2005, 2015). Pre-treatment with the PR extract (at 10 and 100 μg^-1^ ml^-1^) prevented Aβ1–42- and Aβ25–35-induced axonal collapses. Aβ25–35 induced axonal atrophy in cultured neurons (**Figure 5**) as previously shown (Kuboyama et al., 2005). Aβ25–35-induced axonal atrophy was also prevented by simultaneous treatment with the PR extract at doses of 10 and 100 μg^-1^ ml^-1^ (**Figure 5**).

**FIGURE 6:**
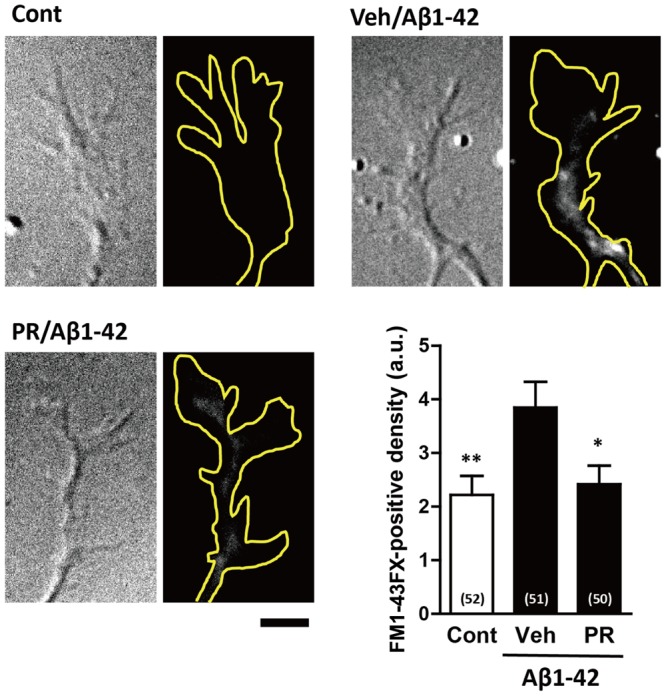
Effects of Polygalae Radix (PR) extract on amyloid β (Aβ)-induced endocytosis. Cortical neurons were cultured for 3 days, and treated with vehicle solution (Cont) or Aβ1–42 (1 μM) for 30 min. Next, they were treated with FM1-43FX (20 μM) for 1 min, and vehicle solution (Cont, Veh) or PR extract (100 μg ml^-1^) for 20 min, before the neurons were fixed. Representative differential interference contrast and fluorescence images are shown. The yellow dotted lines represent outlines of the growth cone. Scale bar, 5 μm. The density of FM1-43FX-positive area in each growth cone were quantified. The mean values of the data are presented together with the standard error. The numbers in parentheses indicate the measured numbers of growth cones. ^∗^*P* < 0.05, ^∗∗^*P* < 0.01 vs. Veh/Aβ1–42; Bonferroni’s multiple comparison test.

Endocytosis is crucial for Aβ-induced axonal collapse and axonal atrophy to occur in cultured neurons (Kuboyama et al., 2015). Additionally, inhibiting Aβ-induced endocytosis is associated with the amelioration of Aβ-induced axonal degeneration and memory deficits *in vivo* (Kuboyama et al., 2015). Therefore, we investigated if PR extract inhibited Aβ-induced endocytosis. To visualize endocytosis, cells were treated with a fluorescent dye, FM1-43FX, which binds to the surface of the cell plasma membrane, and together with the plasma membrane, is endocytosed into the cells. Only the endocytosed FM1-43FX can be visualized following twice wash. The neurons were fixed 20 min after treatment with Aβ1–42, because FM1-43FX was endocytosed into the growth cones, but no axonal collapse was observed at this time-point (Kuboyama et al., 2015). As shown in **Figure 6**, Aβ1–42 increased endocytosis of FM1-43FX at the axonal endings. Pre-treatment with PR extract significantly decreased the density of FM1-43FX-positive staining at axonal endings, indicating that the PR extract prevented endocytosis that was induced by Aβ1–42.

## Discussion

This study was the first to report that the PR extract can prevent cognitive dysfunction when consecutively administered orally to transgenic mice with AD, at an age before the onset of memory deficits. Additionally, the PR extract also inhibited Aβ-induced endocytosis and axon degeneration. Research previously conducted by our group demonstrated that the intracerebroventricular administration of an endocytosis inhibitor, i.e., myristoylated dynamin inhibitory peptide or pitstop 2, prevented Aβ-induced memory deficits (Kuboyama et al., 2015). The effect of the systematic administration of those inhibitors has yet been investigated. Moreover, since many groups are trying to prevent or decrease Aβ accumulation to prevent the progression of AD (Graham et al., 2017), no other group has ever reported a preventive drug for AD that works by inhibiting endocytosis. In contrast, our study shows that the oral administration of PR extract prevented memory deficits without decreasing Aβ deposition in 5XFAD mice. In this study, PR extract was identified as a potent endocytosis inhibitor, which prevented cognitive dysfunction without decreasing Aβ deposits in the mouse model for AD.

Aβ is believed to be a crucial cause for AD (Selkoe and Hardy, 2016). In humans, the level of Aβ in the brain are upregulated before the onset of memory impairment (Jack and Holtzman, 2013). The accumulation of Aβ in the brain is age-dependent, and neurodegeneration is induced before the appearance of memory function impairments. Thus, it can take a few decades for memory impairment to occur in patients with AD. In 5XFAD mice, Aβ plaques and degenerated axons were observed at the age of 2 months and increased in an age-dependent manner (**Supplementary Figure S2**). In contrast, memory impairments only first occurred when the 5XFAD mice were 5 months old (**Supplementary Figure S2**). Memory impairment, in both humans and mice, seems to be evident when Aβ and/or neurodegeneration levels exceed a certain threshold. In the current study, the PR extract inhibited axonal degeneration without decreasing Aβ deposition, thus preserving memory function in the 5XFAD mice. Although many clinical studies have focused on clearing Aβ from the brain, none have succeeded in preserving or recovering memory function in patients with AD (Graham et al., 2017). Since Aβ abundantly accumulates in the brain before the AD symptoms appear (Selkoe and Hardy, 2016), treatment that focuses on clearing Aβ may be belated and insufficient to repair Aβ-induced disruption of neural networks. Therefore, rather than focusing on Aβ, the downstream signaling of Aβ could be a better target for preventing axon degeneration in AD.

Aβ-induced endocytosis can lead to axonal collapse, preventing axonal growth, and consequently causing axonal degeneration (Kuboyama et al., 2015). Endocytosis is thought to reduce the surface area of the plasma membrane and eliminate some essential molecules from the surface of the growth cone (Tojima et al., 2011). After endocytosis of the plasma membrane, the growth cone is unable to maintain its own morphology, and axonal collapse occurs. Aβ also reportedly induced synaptic dysfunction, via endocytosis of α-amino-3-hydroxy-5-methyl-4-isoxazolepropionic acid receptors and *N*-methyl-D-aspartic acid receptors (Snyder et al., 2005; Kurup et al., 2010; Zhao et al., 2010). Amyloid precursor protein on the plasma membrane is endocytosed and cleaved to produce Aβ, which is then released into the interstitial fluid of the brain (Cirrito et al., 2008). Approximately 70% of Aβ in the interstitial fluid was predicted to be produced via the endocytosis pathway (Cirrito et al., 2008). Therefore, the inhibition of endocytosis is certainly a promising target for the prevention of AD.

Tenuifolin, a component of PR, was reported to decrease the secretion of Aβ from COS-7 cells expressing the mutant form of amyloid precursor protein (Lv et al., 2009). Tenuifolin also counteracts Aβ25–35-induced neurotoxicity *in vitro* and *in vivo*, improving spatial memory (Liu et al., 2015). A hydrolysate of a polygalasaponin fraction derived from PR enhanced spatial memory in Aβ25-35-induced amnesic mice, probably via its anti-oxidative activity (Xu et al., 2011). Antioxidants are expected to prevent AD (Vina et al., 2004; Ray et al., 2011a,b). Therefore, these pharmacological effects also possibly contributed to the preventive effects of PR extract in AD. However, these studies were performed only in cultured cells or Aβ-injected animal models. In AD, Aβ deposition and cognitive dysfunction occur in an age-dependent manner. Thus, to examine the preventive activities of drugs against AD in a preclinical study, transgenic animal models of AD, which show AD phenotypes in an age-dependent manner, should be used. To our knowledge, the current study is the first to show the preventive effects of PR against the AD phenotype in a transgenic mouse model of AD.

BT-11, a crude extract of PR, was reported to improve spatial memory in rats suffering from chronic stress or treated with scopolamine (Park et al., 2002; Shin et al., 2009). BT-11 also reportedly enhanced memory function in healthy humans (Lee et al., 2009). PR has traditionally been used in a clinical capacity for more than a thousand years in Japan, China, and South Korea. Therefore, the safety of PR for clinical use has already been established. In our study, the chronic administration of PR extract for 2 months did not influence body weight or general behaviors. Considering that PR has been traditionally been used for memory enhancement, PR extract could be used as a safe preventive drug for AD.

Next, we assessed if the administration of PR extract after the onset of AD pathologies could ameliorate cognitive dysfunction in AD. We previously demonstrated that PR extract promoted axon elongation in cultured neurons after Aβ had already induced axonal degeneration (Naito and Tohda, 2006). Several herbal drugs, which induced axon elongation in neurons damaged by Aβ, also showed the recovery of memory function in aged 5XFAD mice showing memory impairments (Joyashiki et al., 2011; Tohda et al., 2011, 2012; Yang et al., 2017). Tenuifolin and polygalasaponin hydrate derived from PR also led to recovery of memory after Aβ25–35 was injected into the hippocampus of the mice (Xu et al., 2011; Liu et al., 2015). These results indicated that PR extract could putatively enhance the recovery of memory in patients with AD.

In the current study, we clarified the preventive effects of PR extract on axon degeneration and memory impairment in 5XFAD transgenic AD mice, and found a novel pharmacological activity of the PR extract, i.e., the inhibition of endocytosis. As described above, PR extract and its constituents show various pharmacological effects possibly preventing AD. PR extract could be the first multi-functional preventive drug against AD, at least partly due to its ability to inhibit endocytosis.

## Author Contributions

TK and CT designed the experiments and wrote the manuscript. KH, TA, and HY performed and analyzed HPLC experiments. TK performed and analyzed all other experiments.

## Conflict of Interest Statement

The authors would like to disclose that they received research funding from Kobayashi Pharmaceutical Co., Ltd. to conduct this study. The authors declare that the funders had no role in the study design, data collection and analysis, decision to publish or preparation of the manuscript.

## Footnotes

1 http://www.cmd.gov.hk/html/eng/hkcmms/cmmlist.html

## Supplementary Material

FIGURE S1 High performance liquid chromatography (HPLC) profiles. Polygalae Radix (PR) extract and standard compounds were analyzed using two kinds of HPLC protocols, i.e., one to detect tenuifolin **(A,B)** and the other to detect of 3′,6-di-*O*-sinapoyl sucrose ester **(C,D)**. HPLC profiles of PR extract **(A,C)** and standard compounds, tenuifolin **(B)** and 3′,6-di-*O*-sinapoyl sucrose ester **(D)**, are shown. Arrows indicate peaks of tenuifolin **(A,B)** and 3′,6-di-*O*-sinapoyl sucrose ester **(C,D)**, respectively.

FIGURE S2 Age-dependent phenotypes of 5XFAD mice. **(A)** Novel object recognition tests were performed as done in **Figure 2** using WT and 5XFAD male mice at various ages. The numbers in parentheses indicate the age of the mice. Values of preferential indices of each mouse are shown. Paired *t*-test; *n* = 3 mice. **(B,C)** After the behavioral tests, the mice were euthanized and the brain sections were immunostained for phosphorylated neurofilament-H (pNF-H) and amyloid β (Aβ). The density of Aβ plaques **(B)** and degenerated axons associated with the Aβ plaque **(C)** were quantified in the medial prefrontal cortex. The mean values of the data are presented together with the standard error. ^∗∗∗^*P* < 0.001 vs. WT; Bonferroni’s multiple comparison test. *n* = 3 mice.

FIGURE S3 Changes in body weight and exploratory behaviors after the administration of PR extract. Vehicle solution (Veh) or PR extract [at doses of 12 or 60 mg/kg/day; PR (12), PR (60), respectively] were orally administered daily for 56 days to 4-month-old 5XFAD mice. **(A)** The mean values of the body weight of the mice are presented together with the standard error. There is no statistical difference among groups. *P* > 0.05; 2-way repeated measures ANOVA, *n* = 4–5 mice. **(B)** Total number of times the mice encountered the two objects in the open box was counted in the training session. There was no statistical difference among the groups. *P* > 0.05; 1-way ANOVA, *n* = 4–5 mice.

## Abbreviations

Aβ: amyloid β
AD: Alzheimer’s disease
ANOVA: analysis of variance
BSA: bovine serum albumin
HPLC: high performance liquid chromatography
NGS: normal goat serum
PBS: phosphate buffered saline
PFA: paraformaldehyde
pNF-H: phosphorylated neurofilament-H
PR: Polygalae Radix
Veh: vehicle
WT: wild-type
