# Supplementary figures and images for: Polygalae Radix Extract Prevents Axonal Degeneration and Memory Deficits in a Transgenic Mouse Model of Alzheimer’s Disease
Source: Front Pharmacol. 2017 Nov 14;8:805. doi: 10.3389/fphar.2017.00805 (PMC5694549; doi:10.3389/fphar.2017.00805)

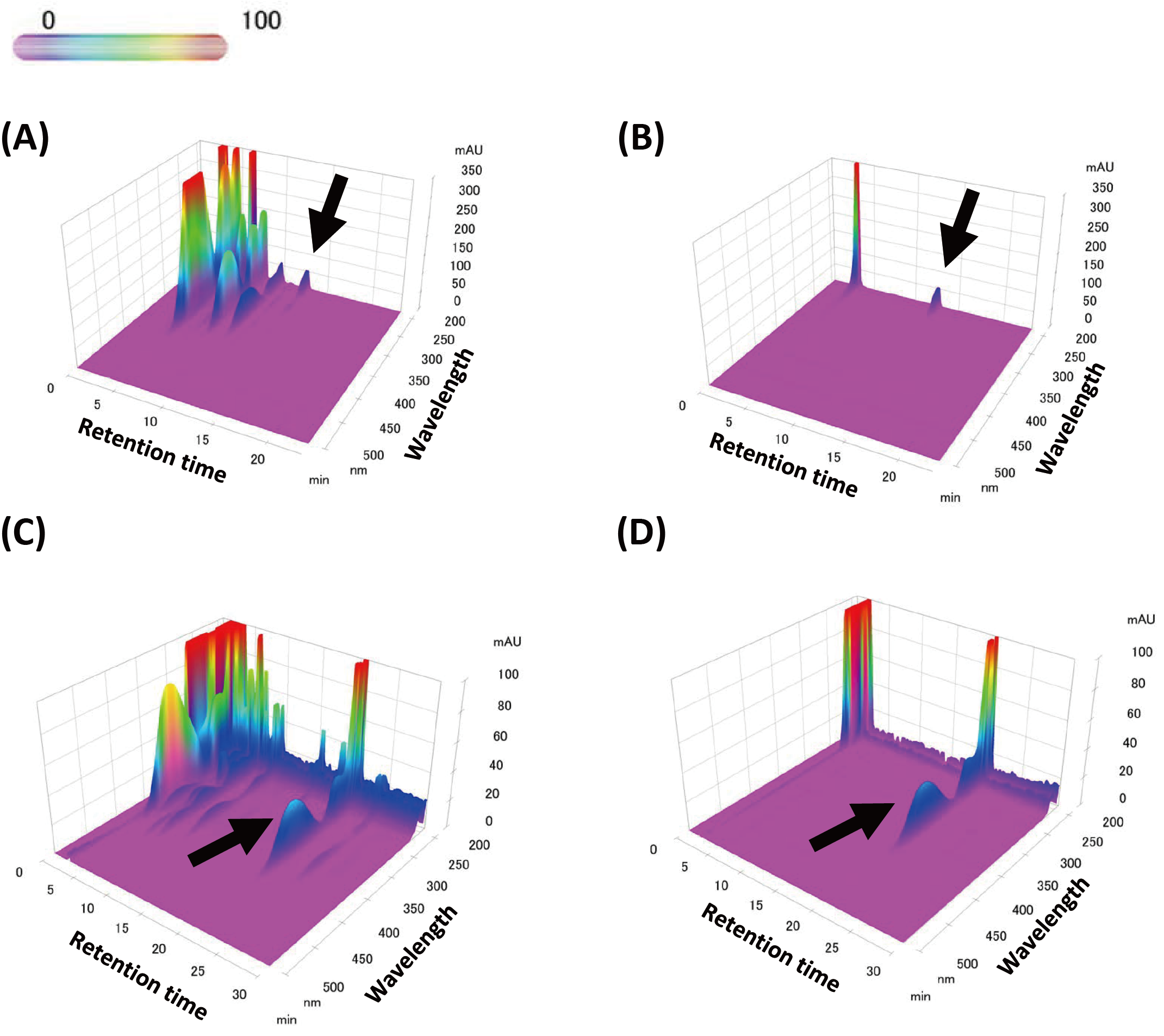

Supplement: FIGURE S1 — High performance liquid chromatography (HPLC) profiles. Polygalae Radix (PR) extract and standard compounds were analyzed using two kinds of HPLC protocols, i.e., one to detect tenuifolin (A,B) and the other to detect of 3′,6-di-O-sinapoyl sucrose ester (C,D). HPLC profiles of PR extract (A,C) and standard compounds, tenuifolin (B) and 3′,6-di-O-sinapoyl sucrose ester (D), are shown. Arrows indicate peaks of tenuifolin (A,B) and 3′,6-di-O-sinapoyl sucrose ester (C,D), respectively. [file Image_1.TIF]

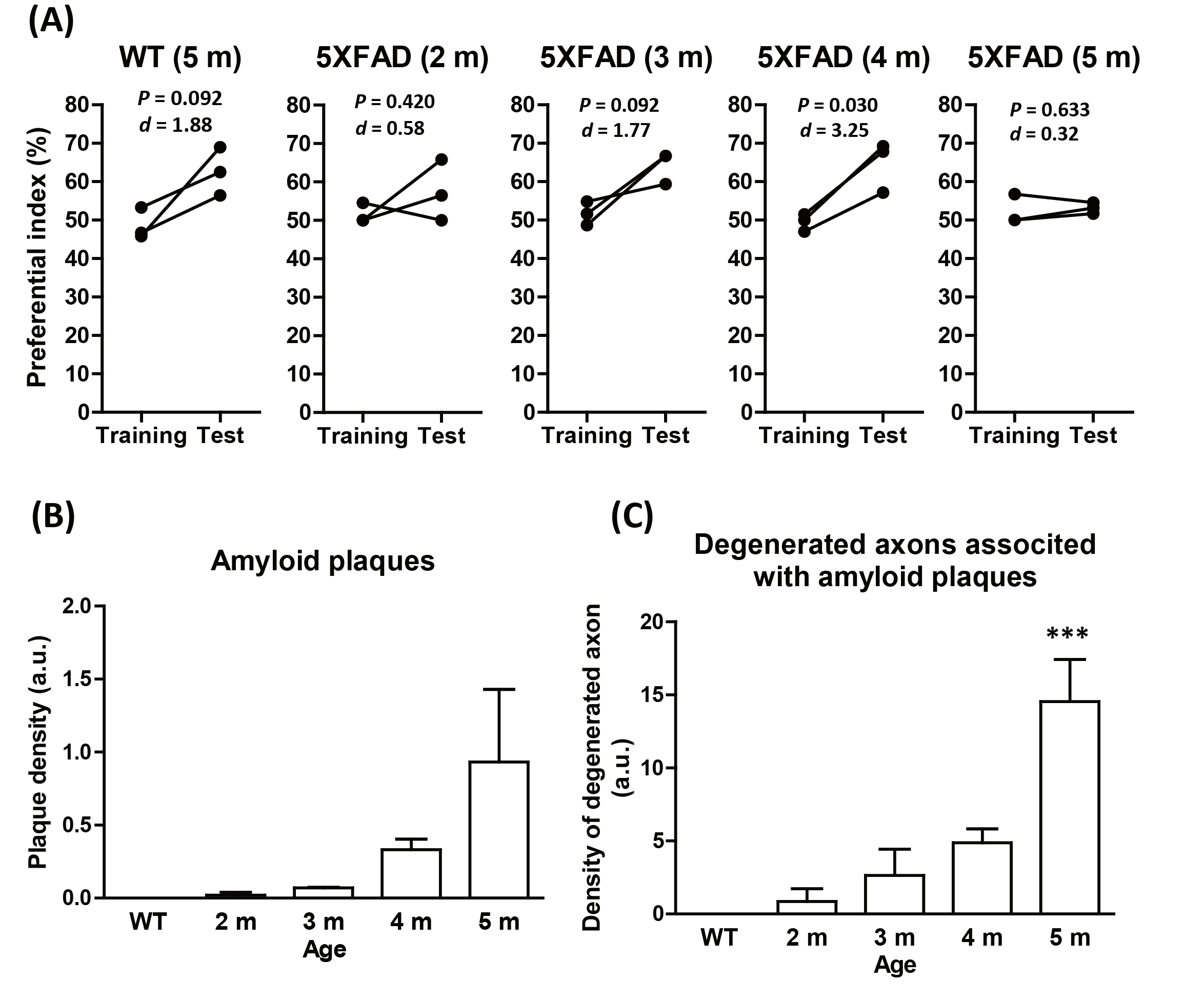

Supplement: FIGURE S2 — Age-dependent phenotypes of 5XFAD mice. (A) Novel object recognition tests were performed as done in Figure 2 using WT and 5XFAD male mice at various ages. The numbers in parentheses indicate the age of the mice. Values of preferential indices of each mouse are shown. Paired t-test; n = 3 mice. (B,C) After the behavioral tests, the mice were euthanized and the brain sections were immunostained for phosphorylated neurofilament-H (pNF-H) and amyloid β (Aβ). The density of Aβ plaques (B) and degenerated axons associated with the Aβ plaque (C) were quantified in the medial prefrontal cortex. The mean values of the data are presented together with the standard error. ∗∗∗P < 0.001 vs. WT; Bonferroni’s multiple comparison test. n = 3 mice. [file Image_2.TIF]

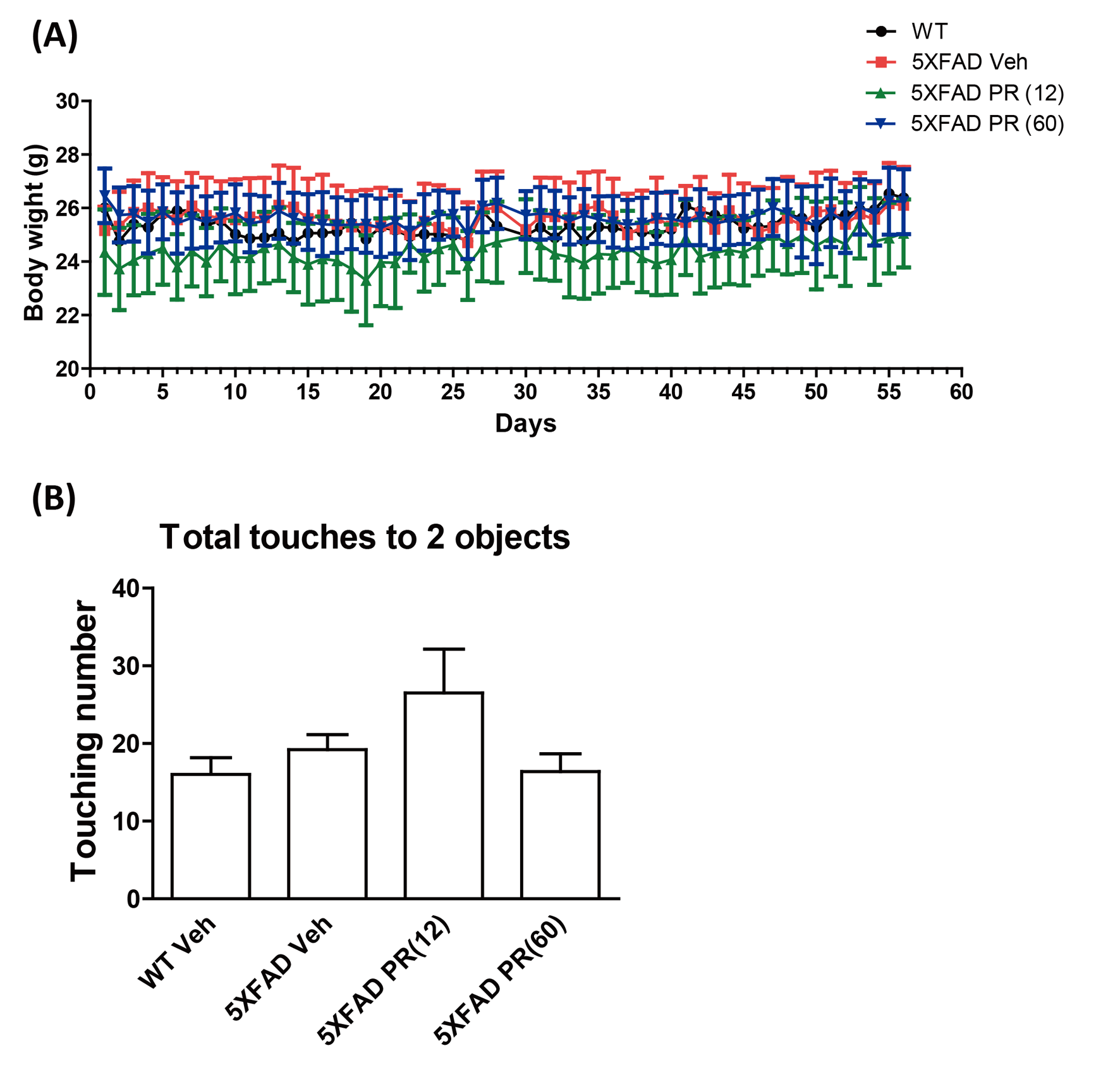

Supplement: FIGURE S3 — Changes in body weight and exploratory behaviors after the administration of PR extract. Vehicle solution (Veh) or PR extract [at doses of 12 or 60 mg/kg/day; PR (12), PR (60), respectively] were orally administered daily for 56 days to 4-month-old 5XFAD mice. (A) The mean values of the body weight of the mice are presented together with the standard error. There is no statistical difference among groups. P > 0.05; 2-way repeated measures ANOVA, n = 4–5 mice. (B) Total number of times the mice encountered the two objects in the open box was counted in the training session. There was no statistical difference among the groups. P > 0.05; 1-way ANOVA, n = 4–5 mice. [file Image_3.TIF]
